# Successful treatment of disseminated intravascular coagulation by recombinant human soluble thrombomodulin in patients with acute myeloid leukemia

**DOI:** 10.1097/MD.0000000000012981

**Published:** 2018-11-02

**Authors:** Miyuki Ookura, Naoko Hosono, Toshiki Tasaki, Kana Oiwa, Kei Fujita, Kazuhiro Ito, Shin Lee, Yasufumi Matsuda, Mihoko Morita, Katsunori Tai, Eiju Negoro, Shinji Kishi, Hiromichi Iwasaki, Takanori Ueda, Takahiro Yamauchi

**Affiliations:** a Department of Hematology and Oncology, Faculty of Medical Science, University of Fukui; b Department of Health and Nutrition, Jin-ai University; c Division of Infection Control, University of Fukui Hospital; d University of Fukui, Japan.

**Keywords:** acute myeloid leukemia, acute promyelocytic leukemia, differentiation syndrome, disseminated intravascular coagulation, thrombomodulin

## Abstract

Disseminated intravascular coagulation (DIC) is a life-threatening condition that frequently occurs in patients with hematologic malignancies. Currently, recombinant human soluble thrombomodulin (rTM) is a therapeutic DIC drug that is manufactured and sold in Japan only. We evaluated the efficacy of rTM compared to that of gabexate mesilate (GM), which was previously used routinely for treating DIC in Japan, in patients with acute myeloid leukemia (AML). This retrospective study enrolled 43 AML patients, including 17 with acute promyelocytic leukemia (APL), that was complicated with DIC. DIC resolution rates in non-APL AML and rTM-treated APL patients were 68.4% and 81.8%, respectively. In non-APL AML patients, the duration of rTM administration was significantly shorter than that of GM (7 vs 11 days), suggesting that rTM could improve DIC earlier than GM, although rTM was used in patients with more severe DIC. Moreover, treatment with rTM significantly improved DIC score, fibrinogen, fibrin/fibrinogen degradation product (FDP), and prothrombin time (PT) ratio. Conversely, treatment with GM only improved the DIC score and FDP. In APL patients, the duration of rTM administration was also significantly shorter than that of GM. No severe side effects associated with the progression of bleeding were observed during rTM administration. These findings suggest that rTM is safe, and its anti-DIC effects are more prompt than GM for treating AML patients with DIC.

## 1 Introduction

Disseminated intravascular coagulation (DIC) is characterized by the excessive activation of coagulation, resulting in fibrin deposition in systemic microvessels. Thrombotic occlusion causes organ failure or severe bleeding owing to the depletion of platelets and coagulation proteins.^[1]^ DIC is a life-threatening condition that frequently occurs in patients with severe sepsis, acute pancreatitis, trauma, solid tumors, and hematologic malignancies.^[1–3]^ Approximately 15% of patients with acute leukemia also have DIC complications.^[4]^ Tissue factor, which activates the extrinsic coagulation pathway, is overexpressed in leukemia cells, causing DIC.^[5]^ Acute promyelocytic leukemia (APL) is a subtype of acute myeloid leukemia (AML) that is characterized by specific biologic and clinical features, including t (15;17)(q22,q12) chromosomal translocation that fuses the promyelocytic leukemia *(*PML*)* gene on chromosome 15 to the retinoic acid receptor-α (RAR*α*) gene on chromosome 17.^[6,7]^ APL patients more frequently present with severe DIC owing to the high expression of tissue factor and annexin II, which activates fibrinolysis.^[8–11]^

Thrombomodulin (TM) is a thrombin receptor on the endothelial cell surface that acts as an important regulator of intravascular coagulation.^[12,13]^ TM traps thrombin to inactivate coagulation, and the thrombin-TM complex activates protein C to form activated protein C (APC), which inactivates factors VIIIa and Va.^[14]^ Thus, TM functions as a negative regulator in response to excess thrombin. Recomodulin (Asahi Kasei Pharma, Tokyo, Japan) is a recombinant human soluble thrombomodulin (rTM), which is similar to physiological TM and can bind to thrombin to inactivate coagulation.^[15]^ In Japan, rTM has been approved as a therapeutic DIC drug since 2008. Over 4000 DIC cases have been treated with rTM in the 2 years following approval (from May 2008 to April 2010),^[16]^ and the post-marketing surveillance revealed the safety and efficacy of rTM.^[17,18]^ In the United States, a randomized, double-blind, placebo-controlled, phase 2b study was performed, which showed evidence that was suggestive of the efficacy of rTM in patients with DIC-associated sepsis.^[19]^

Gabexate mesilate (GM), a synthetic protease inhibitor (SPI), was previously used routinely for treating DIC before rTM was approved in Japan, although no clinical trials have been reported assessing its efficacy for treating DIC.^[20]^ Recent studies have indicated that rTM is more effective than GM for treating DIC in patients with infectious diseases;^[21–23]^ conversely, little research has been conducted among AML patients. Therefore, we retrospectively evaluated the efficacy of rTM compared to that of GM for treating AML patients complicated with DIC.

## 2 Materials and methods

### 2.1 Patients

The retrospective study enrolled 43 patients aged ≥18 years who were identified to have complicated DIC at the onset or during induction chemotherapies for AML between 1992 and 2017 at our institution (University of Fukui Hospital). Patients with DIC mainly caused by infection and patients who did not receive treatment for AML were excluded. AML was diagnosed based on standard morphological evaluations of bone marrow specimens according to the French-American-British (FAB) criteria. APL diagnosis was confirmed based on the distinct morphology of blast cells and the presence of t (15;17)(q22;q12) and/or the PML-RARα fusion gene. Differentiation syndrome (DS), a serious complication in APL patients, was diagnosed based on the presence of fever, weight gain, peripheral edema, interstitial pulmonary infiltrates, pleuropericardial effusion, hypotension, and/or acute renal failure.^[24]^ We collected these data from patients’ electronic medical records, and linkable anonymizing data were saved. The datasets analyzed in the present study are available from the corresponding author upon reasonable request.

### 2.2 DIC diagnosis and treatment

DIC diagnosis was based on the criteria recommended by the Japanese Ministry of Health and Welfare (JMHW), which comprises the presence of underlying disease or organ failure, decreased fibrinogen (Fbg), elevated fibrin/Fbg degradation products (FDPs), and increased prothrombin time (PT) ratio on coagulation tests.^[25]^ DIC was diagnosed when the score exceeded 4 points in patients with leukemia.^[15,26,27]^ GM (FOY, Ono Pharmaceutical co., Ltd., Osaka, Japan, 20–39 mg/kg per day, continuous intravenous infusion)^[20,28]^ and rTM (Recomodulin, 380 U/kg per day, intravenous infusion for 30 min) were used as treatment for DIC. DIC resolution was defined as a DIC score of less than 3 points based on the JMHW diagnosis criteria. The duration of GM or rTM administration was decided by each physician based on the patients’ DIC score. Even if the DIC score was not less than 3 points, rTM and GM were discontinued at each physician's discretion when the DIC score was reduced, and the bleeding tendency was not observed.

### 2.3 Statistical analysis

Wilcoxon signed-rank tests, Mann–Whitney *U* tests, chi-square tests, and log-rank tests were used for statistical analysis. All analyses were performed using the Excel software (ver. 14.5.7, Microsoft), and *P* values <.05 were considered statistically significant.

### 2.4 Ethical conduct of the study

This study was conducted according to the ethical principles outlined in the Declaration of Helsinki, Pharmaceutical Affairs Law of Japan, and other applicable regulations and guidelines. This study was approved by the Institutional Review Board in the medical research support center of the University of Fukui Hospital (reference number 20160047). Informed consent was waived because this study was a retrospective study.

## 3 Results

### 3.1 Comparison of patient characteristics, laboratory data, and treatment outcomes between the rTM and GM groups in non-APL AML patients

The characteristics of non-APL AML patients are presented in Table 1. All 26 patients developed DIC during induction chemotherapies owing to leukemia: 19 patients received rTM (380 U/kg/day) and 7 received GM (20–39 mg/kg/day). No significant difference was observed with respect to age, sex, intensity of induction therapy, complete remission (CR) rate, or survival rate by 28 days between the 2 groups. At diagnosis of leukemia, 5 (26.3%) patients in the rTM group and 2 (28.5%) in the GM group had bleeding symptoms such as purpura (n = 3 in the rTM group and n = 1 in the GM group), gingival bleeding (n = 1 in the rTM group and n = 1 in the GM group), and hemorrhagic cerebral infarction (n = 1 in the rTM group). These patients were diagnosed as severe because they were >grade 2 according to the National Cancer Institute Common Terminology Criteria for Adverse Events. These bleeding symptoms, except hemorrhagic cerebral infarction, improved during rTM, or GM treatment. In the rTM group, 2 patients died during induction therapies; 1 had hemorrhagic cerebral infarction at AML diagnosis and died on day 14 owing to the progression of intracerebral bleeding despite DIC improvement. Another patient died owing to septic shock on day 10. In the GM group, 1 patient died owing to pulmonary alveolar hemorrhage on day 20, although DIC had resolved. No severe side effects associated with the progression of bleeding were observed during rTM or GM administration.

**Table 1:**
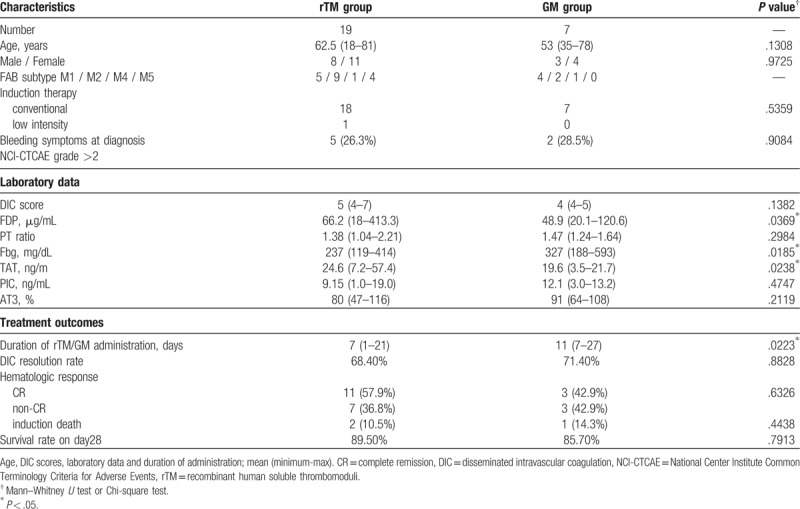
Comparison of clinical characteristics, laboratory data, and treatment outcomes between rTM and GM groups in non-APL AML patients.

### 3.2 Efficacy of rTM compared with that of GM in non-APL AML patients

**Figure 1:**
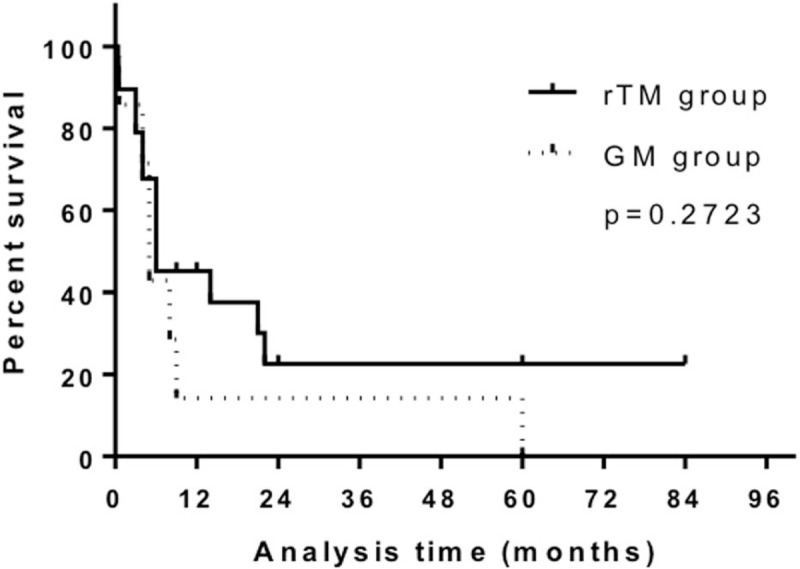
Kaplan–Maier estimates of overall survival according to the therapy used for disseminated intravascular coagulation in non-acute promyelocytic leukemia acute myeloid leukemia patients.

The DIC resolution rates were 68.4% and 71.4% in the rTM and GM groups, respectively (Table 1). No significant differences in the DIC resolution rates and overall survival (OS) were noted; however, the survival curve in the GM group was below that in the rTM group (Fig. 1).

**Figure 2:**
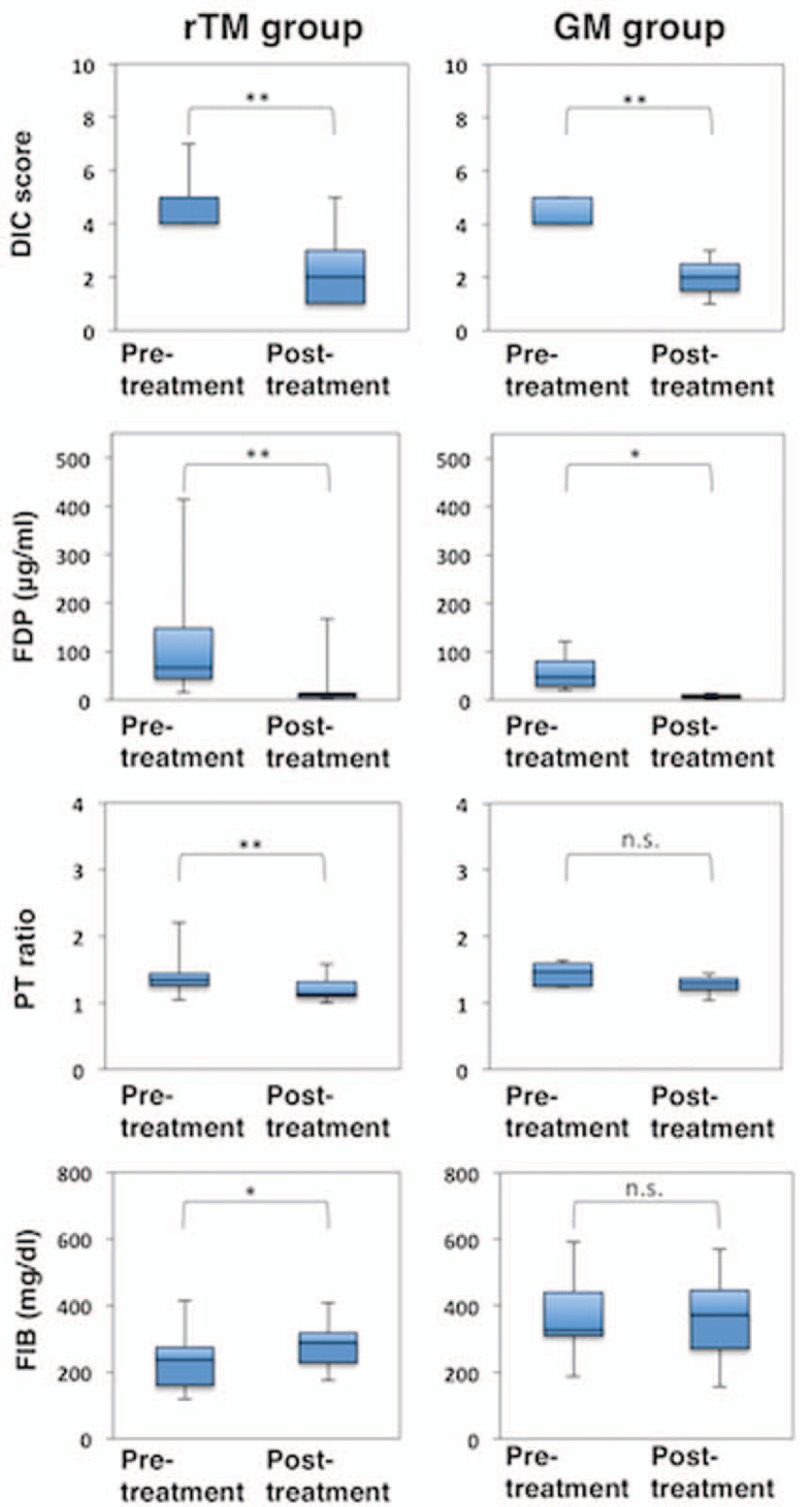
Efficacy of recombinant human soluble thrombomodulin for treating patients with non-acute promyelocytic leukemia acute myeloid leukemia. Disseminated intravascular coagulation scores and hemostatic molecular markers before (pre-treatment) and after (post-treatment) the administration of recombinant human soluble thrombomodulin. The box presents the interquartile range (25th–75th percentile), and the line within the box is the median value. ∗*P* < .05, ∗∗*P* < .01; n.s.; not significant, Wilcoxon signed-rank tests.

The scores of coagulation markers, such as FDP, Fbg, and thrombin-antithrombin complex (TAT), were significantly worse in the rTM group; however, the duration of rTM administration was significantly shorter than that of GM (7 vs 11 days, *P* = .0223, Table 1). Treatment with rTM significantly improved the DIC score (*P* < .0001), FDP levels (*P* = .0032), PT ratio (*P* = .0037), and Fbg levels (*P* = .0398, Fig. 2). Conversely, treatment with GM only improved the DIC score (*P* = .0013) and FDP levels (*P* = .0183).

### 3.3 Comparison of patient characteristics, laboratory data, and treatment outcomes between the rTM and GM groups in APL patients

The characteristics of APL patients are presented in Table 2. All 17 patients developed DIC during all-trans retinoic acid (ATRA)-based induction regimens; 11 patients received rTM (380 U/kg/day) and 6 received GM (20–39 mg/kg/day). At our institution, rTM was administered to all APL patients with DIC after rTM was approved for marketing. All 6 patients treated with GM were cases before rTM approval. No significant difference was observed in age, sex, therapeutic regimens, laboratory data, CR rate, or survival rate by 28 days between the 2 groups. At diagnosis of leukemia, 7 (63.6%) patients in the rTM group and 4 (66.7%) in the GM group had bleeding symptoms such as purpura (n = 4 in the rTM group and n = 3 in the GM group), gingival bleeding (n = 1 in the rTM group and n = 1 in the GM group), hematoma (n = 1 in the rTM group), and mild subarachnoid hemorrhage (n = 1 in the rTM group), all of which were diagnosed as severe. No severe side effects associated with the progression of bleeding were observed, and all these symptoms resolved during rTM or GM administration.

**Table 2:**
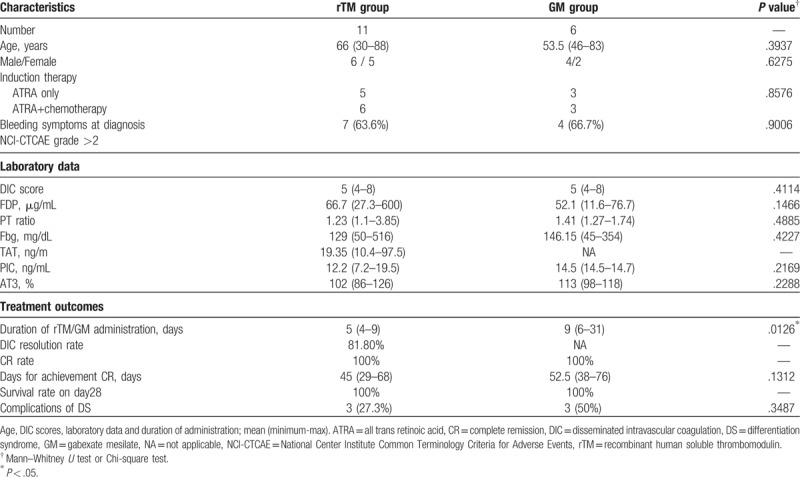
Comparison of clinical characteristics, laboratory data, and treatment outcomes between rTM and GM groups in APL patients.

### 3.4 Efficacy of rTM compared with that of GM in APL patients

**Figure 3:**
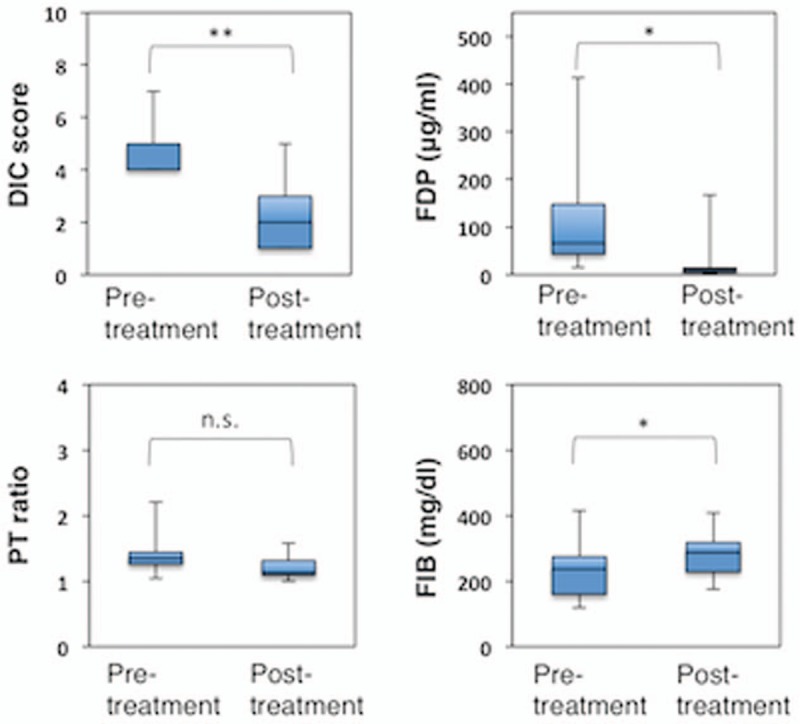
Efficacy of recombinant human soluble thrombomodulin for treating patients with acute promyelocytic leukemia. Disseminated intravascular coagulation scores and hemostatic molecular markers before (pre-treatment) and after (post-treatment) the administration of recombinant human soluble thrombomodulin. The box presents the interquartile range (25th–75th percentile), and the line within the box is the median value. ∗*P* < .05, ∗∗*P* < .01; n.s.; not significant, Wilcoxon signed-rank tests.

In the rTM group, the DIC resolution rate was 81.8%, and it was accompanied with significant improvements in FDP (*P* = .0209), Fbg (*P* = .0309), and DIC scores (*P* < .0001, Table 2Fig. 3). A reduction in the DIC score from baseline was observed in all patients in the rTM group (median reduction, 3 points). Similar to non-APL AML patients, the duration of rTM administration was significantly shorter than that of GM in APL patients (5 vs 9 days, *P* = .0126). We also assessed days of CR achievement and frequency of DS. A median of 45 and 52.5 days were required to achieve CR in the rTM and GM groups, respectively (*P* = .1312).

**Figure 4:**
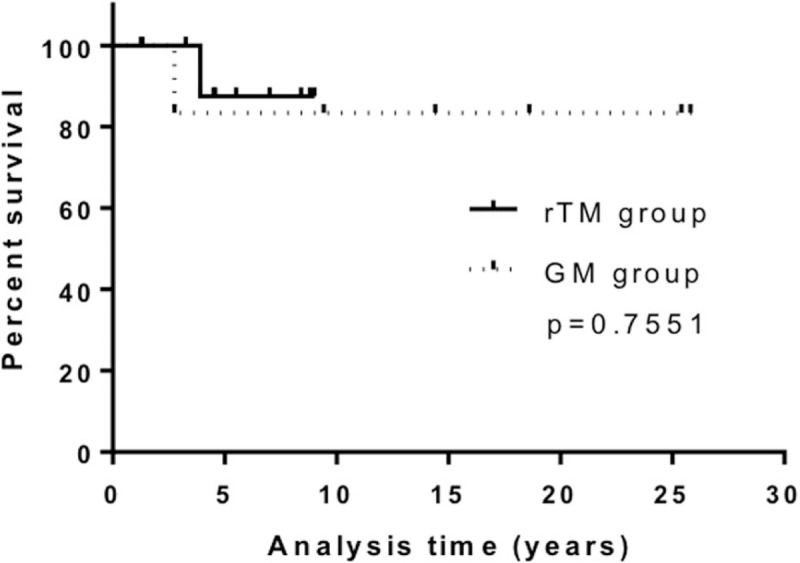
Kaplan–Maier estimates of overall survival according to the therapy used for disseminated intravascular coagulation in acute promyelocytic leukemia patients.

DS occurred in a median of 16 (range 12–17) days after initiating ATRA treatment, and the median white blood cell count was 3600/μL (overall range 1700–6400/μL) at diagnosis of DS. The frequency of DS was 27.3% and 50.0% in the rTM and GM groups, respectively (*P* = .3487). There was no significant difference in survival between the 2 groups based on whether patients were treated with rTM or GM (Fig. 4).

## 4 Discussion

OS in AML patients with DIC is significantly worse than that in AML patients without DIC.^[29]^ Thus, in addition to controlling AML, successful treatment of DIC itself is indispensable for better clinical outcomes.

In this study, DIC resolution rates for patients with non-APL AML and APL treated with rTM were 68.4% and 81.8%, respectively. Previous studies have reported that these resolution rates were 55.1% (n = 216) and 57.8% (n = 135), respectively,^[17]^ thus at our institution, the DIC resolution rates were markedly better than that previously reported. For infectious diseases, some reports indicated that rTM was more effective^[20,23]^ and that it significantly reduced mortality and increased survival compared with GM.^[22,30,31]^ In the setting of hematologic malignancies, Takezako et al revealed that OS was superior in AML patients treated with rTM when compared with heparin.^[29]^ Aota et al reported that there were no significant differences in the DIC resolution rates and mortality between the rTM (n = 21) and GM (n = 206) groups.^[22]^ In the present study, rTM treatment could contribute to improved OS in non-APL AML patients compared with GM treatment. However, further studies are warranted to further clarify this relationship.

In non-APL AML patients, the scores of coagulation markers, such as FDP, Fbg, and TAT, were significantly worse in the rTM group before DIC treatment; however, the duration of rTM administration was significantly shorter than that of GM. Furthermore, treatment with rTM more significantly improved FDP, PT ratio, and Fbg compared with GM. These findings suggest that rTM has more potent anti-DIC effects and improves DIC earlier than GM. In APL patients, Kawano et al reported that the DIC resolution rate was better in the rTM group (n = 6, 66.6%) than in the SPI group (n = 4, 25.0%).^[32]^ In this study, although the DIC resolution rate did not significantly differ, the duration of rTM administration was significantly shorter than that of GM in APL patients.

DS, formerly known as retinoic acid syndrome, is a common and potentially severe complication observed in APL patients undergoing induction therapy with ATRA.^[33]^ Although DS pathogenesis has not been completely clarified, it has been speculated that ATRA-induced differentiation of leukemic blasts and promyelocytes leads to the release of cytokines, systemic inflammatory response syndrome, endothelium damage with capillary leak syndrome, occlusion of circulation in micro vessels, and tissue infiltration.^[34–36]^ The problem seems to lie in the fact that there is little evidence of treatment for DS prevention and management.^[36]^ One study revealed that in a clinical situation, when physicians suspect DS development preemptive treatment with dexamethasone should be immediately initiated because DS is associated with increased morbidity and mortality during induction therapy.^[33]^ TM possesses anti-DIC effects and anti-inflammatory effects owing to its N-terminal lectin-like domain of TM.^[37–40]^ Moreover, TM-APC has anti-inflammatory effects.^[37,41–44]^ The lectin-like domain sequestrates and degrades high-mobility group-B1 protein, which is a pro-inflammatory molecule that stimulates production of various inflammatory cytokines.^[37,45]^ Similarly to TM, rTM has these structures, and it can be presumed that rTM contributes to DS prevention via its anti-inflammatory effects.^[46]^ In the present study, DS development tended to be less frequent in the rTM group compared with that in the GM group (27.3% vs 50%). rTM might be preferable for treating APL because its anti-inflammatory effect may prevent DS development. Additionally, Ikezoe et al reported that TM enhances anti-leukemic effects of ATRA in APL cells;^[47]^ Kawano et al also suggested that APL patients treated with rTM tended to demonstrate molecular CR.^[32]^ Our findings also showed that the rTM group tended to achieve CR earlier than the GM group. Therefore, the combination rTM and ATRA may show more potent anti-tumor effects in APL patients.

Our study had several limitations. First, we had a small sample size, and different numbers of patients were enrolled in each group. Additionally, this was a retrospective study that included patients treated over a decade ago; this might have affected the validity of our results.

In conclusion, our study indicated the beneficial effects of rTM for AML patients. Importantly, the anti-DIC effects of rTM is prompter than GM for treating non-APL AML patients. In addition, the other effects of rTM, with the exception of the anti-DIC, anti-inflammatory, and anti-tumor effects, might be preferable for APL patient outcomes. In the future, a large randomized trial is needed to determine the advantages of rTM treatment for DIC in AML patients. However, our findings will contribute to the better understanding of the efficacy of rTM.

## Author contributions

**Data curation:** Toshiki Tasaki.

**Investigation:** Kana Oiwa, Kei Fujita, Kazuhiro Ito, Shin Lee, Yasufumi Matsuda, Mihoko Morita, Katsuhiro Tai, Eiju Negoro, Shinji Kishi, Hiromichi Iwasaki, Takanori Ueda.

**Writing – original draft:** Miyuki Ookura.

**Writing – review & editing:** Naoko Hosono, Takahiro Yamauchi.
